# Systematic review of outcome measures following chemoradiotherapy for the treatment of anal cancer (CORMAC)

**DOI:** 10.1111/codi.14103

**Published:** 2018-04-17

**Authors:** R. Fish, C. Sanders, N. Ryan, S. Van der Veer, A. G. Renehan, P. R. Williamson

**Affiliations:** ^1^ Division of Cancer Sciences School of Medical Sciences Faculty of Biology, Medicine and Health University of Manchester Manchester UK; ^2^ Peritoneal and Colorectal Oncology Centre Christie NHS Foundation Trust Manchester UK; ^3^ Centre for Primary Care University of Manchester Manchester UK; ^4^ Division of Cancer Sciences School of Medical Sciences Faculty of Biology, Medicine and Health Fifth Floor ‐ Research St Mary's Hospital University of Manchester Manchester UK; ^5^ Centre for Health Informatics Informatics, Imaging and Data Science School of Health Sciences Faculty of Biology, Medicine and Health University of Manchester Manchester UK; ^6^ Farr Institute of Health Informatics Research Health eResearch Centre University of Manchester Manchester UK; ^7^ MRC North West Hub for Trials Methodology Research Department of Biostatistics University of Liverpool Liverpool UK

**Keywords:** Anal cancer, radiotherapy, outcomes, core outcome sets, trials methodology

## Abstract

**Aim:**

Six Phase III randomized trials have determined the effectiveness of chemoradiotherapy as primary treatment for anal squamous cell carcinoma (ASCC), but outcomes reported in these trials varied widely, hindering evidence synthesis. To improve reporting in all future trials, we aim to develop a core outcomes set (COS). As the first stage of COS development, we undertook a systematic review to summarize the outcomes reported in studies evaluating chemoradiotherapy for ASCC.

**Method:**

Systematic literature searches identified studies evaluating radiotherapy or chemoradiotherapy for ASCC. Outcomes and accompanying definitions were extracted verbatim and categorized into domains.

**Results:**

From 5170 abstracts, we identified 95 eligible studies, reporting 1192 outcomes and 533 unique terms. We collapsed these terms into 86 standardized outcomes and five domains: survival; disease activity; life impact [including quality of life (QoL)]; delivery of care; and toxicity. The most commonly reported domains were survival and disease activity, reported in 74 (86%) and 54 (62%) studies, respectively. No outcome was reported in every publication. Over half (43/86) of the standardized outcome terms were reported in fewer than five studies, and 21 (25%) were reported in a single study only. There was wide variation in definitions of disease‐free survival, colostomy‐free survival and progression‐free survival (PFS). Anal continence was reported in only 35 (41%) studies.

**Conclusion:**

Outcomes reported in studies evaluating chemoradiotherapy for ASCC were heterogenous and definitions varied widely. Outcomes likely to be important to patients, such as ano‐rectal function, toxicity and QoL, have been neglected. A COS for future trials will address these issues.

## Introduction

Anal squamous cell carcinoma (ASCC) is a human papillomavirus (HPV)‐related malignancy 1, the incidence of which has increased two‐ to three‐fold in many populations 2, including the UK 3, in the last three decades. In the past 20 years, six Phase III randomized controlled trials (RCTs) 4, 5, 6, 7, 8, 9 determined the effectiveness of chemoradiotherapy as primary treatment in patients with ASCC, and it is now the primary treatment for 75–80% of patients 10. Locoregional failure occurs in 18–25% of patients and requires radical salvage surgery involving multidisciplinary approaches 11, 12. Overall, treatment‐related morbidity is thought to be considerable, but generally is poorly quantified. Five‐year overall survival in patients with ASCC, treated by chemoradiotherapy, is approximately 75% 10; it is therefore likely that many survivors are living with treatment‐related side‐effects.

While the above six trials 4, 5, 6, 7, 8, 9 determined effectiveness, the outcome measures reported in these studies varied widely (Table 1). Even among trials that included local disease control as the primary outcome, definitions of this outcome varied. There was greater consistency in reporting secondary outcomes across these trials, but the emphasis has been survival and disease activity outcomes. None of the trials comprehensively addressed long‐term side‐effects or quality of life (QoL), suggesting that outcomes which reflect issues likely to be important to patients are under‐represented. A recent narrative review from Glynne‐Jones *et al*. 13, critiquing Phase III randomized trials of interventions for ASCC, echoed these summaries and concluded that the ‘quality of outcome reporting in RCTs of squamous cell carcinoma of the anus is inconsistent’.

**Table 1:** Primary and secondary outcomes in six Phase III randomized controlled trials of chemoradiotherapy interventions for ASCC

Trial Authors, year of publication	Local treatment failure	Progression‐free survival	Disease‐free survival	Colostomy‐free survival	Colostomy	Acute toxicity	Overall survival	Cancer‐specific survival	Local/regional control
ACT I (1996) 5	✓^1^			♦			♦	♦	
RTOG 87‐04 (1996) 7	✓^2^		♦	♦	♦	♦	♦		♦
EORTC (1997) 6	✓^3^				♦	♦	♦		
RTOG 98‐11 (2008) 4			✓		♦	♦	♦		♦
ACCORD‐03 (2012) 9				✓			♦	♦	♦
ACT II (2013) 8	✓^4^	✓		♦		✓	♦	♦	♦

✓Primary outcome; ♦ Secondary outcome; 1, clinically, at 6 weeks; 2, on biopsy, post‐irradiation; 3, clinically, at 6 weeks; 4, clinically, at 26 weeks; ASCC, anal squamous cell carcinoma.

Poor selection of outcome measures in clinical trials reduces the quality and relevance of the results 14, and inconsistent outcome selection between trials in the same health area hinders evidence synthesis 14. Both of these issues can be addressed through the use of a core outcome set (COS), ‘an agreed, standardized set of outcomes used in all trials in a particular health area that is developed employing rigorous consensus methods involving all key stakeholders’ 15. The benefits of a COS are increasingly recognized by research funding bodies, regulators and journal editors, via the work of the Core Outcome Measures in Effectiveness Trials (COMET) initiative 16.

A new international trial involving anal cancer patients, PLATO (PersonaLizing Anal cancer radioTherapy dOse, incorporating ACT3, ACT4 and ACT5) 17, commenced recruitment in the UK in 2017. To better inform PLATO and other future anal cancer trials, we are developing a COS for trials of chemoradiotherapy for ASCC. The scope of this COS will be limited to trials of chemoradiotherapy interventions because chemoradiotherapy is the primary intervention in the majority of patients with ASCC and PLATO is the only major trial in this field at the current time. Surgical therapy and chemoradiotherapy will have relevant outcomes in common but there are likely to be a significant number of outcomes unique to each modality (e.g. surgical complications; radiotherapy toxicities). A defining feature of a COS is that it is a list of outcomes that should be measured and reported in *all* trials addressing a specific health condition. A COS that aimed to be relevant to both radiotherapy with or without chemotherapy and surgical intervention would have to be limited and only include the outcomes directly relevant to both these modalities. Furthermore, it may result in exclusion of outcomes that are of key importance to the majority who undergo primary chemoradiotherapy without surgery.

This systematic review aims to summarize the outcomes reported in studies evaluating chemoradiotherapy for ASCC as the preliminary step in the development of the COS.

## Method

The protocol for this systematic review was registered prospectively with the International Prospective Register of Systematic Reviews (PROSPERO CRD42016036540) 15. We report our review in accordance with PRISMA guidelines 18.

### Search strategy

MEDLINE, Embase, Cochrane CENTRAL and Cumulative Index to Nursing and Allied Health Literature (CINAHL) databases were searched from their commencements until December 2016 to identify studies evaluating radiotherapy or chemoradiotherapy interventions in patients with ASCC. A search strategy for MEDLINE was designed following discussions with the Cochrane Colorectal Cancer Review Group, then adapted for the other databases. Searches were limited to studies, published in English, of adults. Complete search strategies are available through the PROSPERO registry. Reference lists of included studies were hand‐searched for additional relevant studies.

### Study eligibility and data extraction

RCTs, prospective and retrospective cohort studies, systematic reviews and cross‐sectional studies of radiotherapy or chemoradiotherapy, as initial treatment in adults with ASCC, were included. Studies of treatment not including radiotherapy, of purely palliative interventions, or of second‐line or subsequent treatment, were excluded. Qualitative studies were included to capture issues identified as important to patients. However, qualitative methodology does not require that outcomes are defined *a priori* or accompanied by a definition; therefore, outcomes identified were included in the outcome long‐list for COS development but excluded from the descriptive analysis in this paper. Outcomes specified in the objectives of systematic reviews were extracted as it is possible for the authors of a systematic review to specify novel outcomes (e.g. a new composite outcome that could be derived from individual outcomes reported in the existing literature). To prevent duplication, outcomes were not extracted from the individual studies included in systematic reviews.

Where multiple papers were found reporting the same data, only the earliest article was included. Articles reporting long‐term results of eligible studies were included alongside the original publication to ensure that any additional outcomes were captured. Titles and abstracts were collated and duplicates removed with Endnote X7 (Thompson Reuters, New York, NY, USA). All abstracts were screened independently by two researchers (RF and NR) against eligibility criteria. Disagreements were resolved by discussion between reviewers. Full‐text screening was undertaken by a single researcher (RF).

Data were extracted from full‐text articles by two researchers (RF and SV) using a Microsoft Access data‐collection form designed and piloted before commencing data collection. Detailed guidance notes accompanied the data‐collection forms. Baseline data for each study were collected, including study design, intervention, and number of participants. All outcomes were extracted verbatim with any accompanying definition, the start point (for time‐to‐event outcomes) and section location within the article (e.g. listed in methods/results section). Where outcomes were defined by a citation, the definition was extracted verbatim from the cited article. Details of all outcome measurement instruments were collected. The individual outcomes measured within each health‐related QoL (HRQoL) tool were extracted following principles set out by Macefield 19.

### Assessment of bias

As there will be no synthesis of results data in this systematic review, no assessment of the methodological quality of the included studies was performed.

#### Cataloguing of outcomes and domain categorization

Verbatim outcomes were initially reviewed by a single researcher (RF) and assigned a standardized name (‘standardized outcome term’) to overcome variations in wording used for the same outcome (a full list of verbatim outcomes and standardized outcome terms is listed in Table S1).

The list of standardized outcome terms was reviewed and each item was assigned to one of five outcome domains: survival; disease activity; delivery of care; life impact; and toxicity. Toxicity was further subdivided according to body system. The outcome domains were defined following a systematic review of outcome categories used in cancer COS development projects, and mapped to the standardized taxonomy for outcomes proposed by the COMET initiative 20. The standardized outcome term and domain assigned to each verbatim outcome were reviewed and agreed at a meeting of the CORMAC Study Advisory Group (SAG), composed of experts in the field of anal cancer, including clinical oncologists, a colorectal surgeon, radiologist, clinical trials methodology expert, qualitative research expert and a patient partner.

Time‐varying and time‐to‐event outcomes (survival, disease activity and delivery of care outcomes) are captured in follow‐up studies (randomized and nonrandomized trials; prospective and retrospective cohort studies) but cannot be measured in cross‐sectional studies. These two types of study design were therefore described separately for analysis.

## Results

### Studies identified

**Figure 1:**
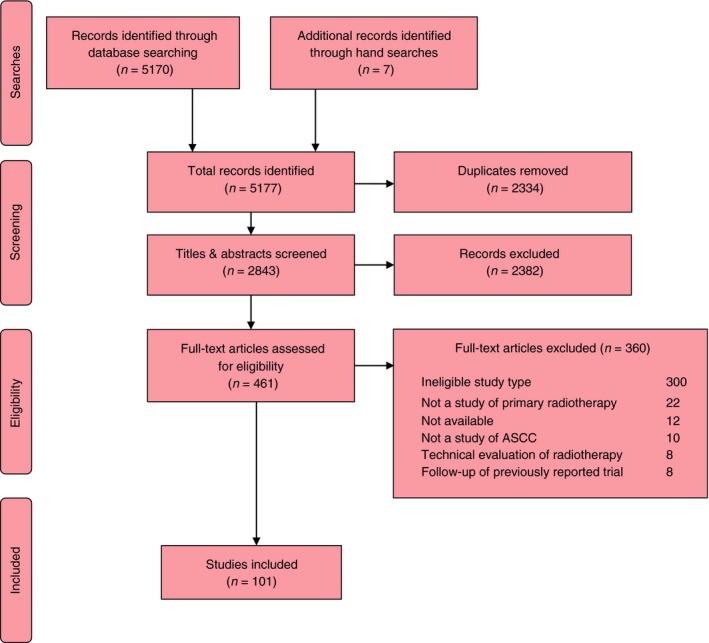
Preferred Reporting Items for Systematic Reviews (PRISMA) diagram of studies. ASCC, anal squamous cell carcinoma.

From 5170 abstracts, and following exclusion of 2334 duplicates, we screened 461 full‐text papers and identified 94 eligible studies. Citation searching identified a further seven studies, totalling 101 studies with 9147 participants (Fig.**** 1).

Summary characteristics of the included studies are listed in Table S2. Of the 101 studies, two‐thirds (66 studies) were published after 2000; 69 were cohort studies and five evaluated outcomes after radiotherapy alone.

Four systematic reviews and two qualitative studies were included for outcome longlisting for COS development but were excluded from descriptive analysis and therefore are not considered further in this paper. Of the remaining 95 papers, 86 follow‐up studies were included in the analysis of time‐varying and time‐to‐event outcomes. Nine cross‐sectional studies were additionally included for analysis of HRQoL and toxicity outcomes.

### Outcomes reported

In total, 1192 individual outcomes were extracted verbatim from the 95 included studies. There were 533 unique terms collapsed into 86 ‘standardized outcome terms’, representing outcomes with the same meaning but with differing wording, and assigned to the appropriate outcome domain (full list in Table S1). Eighty‐six follow‐up studies reported outcomes across all domains. Cross‐sectional studies (*n* = 9) reported outcomes in the toxicity and life impact domains only.

There were 1151 outcomes reported in the 86 longitudinal studies, consolidated into 83 standardized outcome terms. Disease activity and survival were the most common outcome domains in 81 (94%) and 78 (91%) studies, respectively (Table**** 2). Life impact outcomes were reported in only five (6%) studies, and sexual and reproductive toxicity and constitutional symptoms (for example, fatigue) were reported in 11 (14%) and 12 (14%) studies, respectively.

**Table 2:** Outcome domains identified and how they were reported in 86 follow‐up studies

Outcome domains	Studies	Individual outcomes	Unique outcome terms	Standardized outcome terms
Survival	78	191	33	11
Disease activity	81	249	111	7
Life impact	5	7	1	1
Delivery of care	11	14	12	1
Toxicity				
Gastrointestinal	72	317	151	22
Dermatological	57	88	53	6
Haematological	48	120	39	6
Musculoskeletal	14	19	18	3
Urinary	33	45	26	6
Sexual and reproductive	11	15	15	6
Constitutional symptoms	12	18	10	6
Other toxicities^a^	35	68	51	8
Total	86	1151	520	83

Values are given as absolute numbers.

a Cardiac, lymphatic, immunological, respiratory, hepatic, neurological, endocrine and non‐specified toxicity.

Over half (43 of 83) the standardized outcome terms were reported in fewer than five studies (Table S3), with 21 (25%) reported in a single study only. Outcomes reported in only one study include stoma complications, urinary incontinence, and erectile dysfunction.

Nine cross‐sectional studies reported 21 outcomes exclusively in the toxicity and life impact domains; all reported global HRQoL; five studies reported gastrointestinal toxicity; urinary and sexual and reproductive toxicity were each reported in a single study.

### Evaluation of outcome definitions

Of 1151 outcomes identified in the 86 longitudinal studies, 617 (54%) were accompanied by a definition. All 21 outcomes in the nine cross‐sectional studies were accompanied by a definition.

### Disease activity

The most commonly reported outcome in this domain was treatment response in 42 (49%) of the 86 follow‐up studies, with a definition provided in 23 studies. Verbatim terms used include clinical response, pathological response, tumour response, tumour regression and regression or response without further specification. Definitions used incorporated criteria for grading the level of response; modality of assessment; and timing of assessment and there was heterogeneity in all of these aspects (Table S4). Twenty‐two different definitions were used in the 23 studies; the most commonly used criteria for assessing the level of response was the Response Evaluation Criteria In Solid Tumours (RECIST), used in six studies 8, 21, 22, 23, 24, 25. However only two of the studies using RECIST assessed response at the same time points 26, 27.

### Survival

A survival or composite survival outcome was reported in 81 follow‐up studies; the most common was overall survival in 73 (84%) studies, with a definition provided in 28 of these. Disease‐specific survival was reported in 23 (27%) studies and defined in eight, with three different definitions: death from disease 21, 28, 29; death from disease or death with disease present 30, 31, 32, 33; or death from disease or complications of disease 9.

A composite survival outcome was reported in 56 studies. The most common composite survival outcomes were disease‐free survival in 34 (33%) studies and colostomy‐free survival reported in 35 (34%) studies. Where disease‐free survival was reported, the outcome was defined in 20 studies, using nine different definitions (Table**** 3). The most commonly used definition (local, regional or distant failure, second primary or death from any cause) was used in five studies 4, 7, 34, 35, 36. Four of these studies are from the same research organization (Radiation Therapy and Oncology Group) including two papers reporting short‐ and long‐term results of the same study 4, 34.

**Table 3:** Events specified in definitions of disease‐free survival

Author (year) [Ref.]	Relapse/recurrence NOS	Progressive disease	Local relapse/failure	Regional relapse/failure	Distant relapse/failure	Second primary	Cancer‐related death	Death from any cause
Ceresoli *et al*. (1998) 47 El‐Hadaad *et al*. (2015) 23 Provencher *et al*. (2010) 48	✓							✓
Franco *et al*. (2015) 28 Peiffert *et al*. (2012) 9	✓						✓	
Mendenhall *et al*. (1996) 49 Hu *et al*. (1999) 50 Simpson *et al*. (2012) 51	✓							✓
Vendrely *et al*. (2015) 52		✓						✓
Eng *et al*. (2014) 53			✓		✓		✓	
Yates *et al*. (2015) 54 Sischy *et al*. (1989) 55 Moureau‐Zabotto *et al*. (2011) 56			✓		✓			✓
Kichenadasse *et al*. (2007) 57			✓	✓				✓
Chuong *et al*. (2012) 58			✓	✓	✓			✓
Gunderson *et al*. (2013) 34 Yeung *et al*. (2014) 35 Ajani *et al*. (2008) 4 Konski *et al*. (2008) 36 Flam *et al*. (1996) 7			✓	✓	✓	✓		✓

NOS, not otherwise specified.

Where colostomy‐free survival was reported, the outcome was defined in 23 (of 35); varying from any colostomy event in 15 studies to those using additional inclusion criteria for colostomy events, such as colostomy within a specific time period or for a specific indication (seven studies). All seven studies in the latter category used different additional inclusion criteria (Table**** 4).

**Table 4:** Definitions of colostomy‐free and progression‐free survival

Author (year) [Ref.]	Definition
	**Colostomy‐free survival**
James *et al*. (2013) 8	All pretreatment colostomies not reversed within 8 months after starting treatment or any colostomies after treatment
Peiffert *et al*. (2012) 9	Colostomy for progression, relapse or complication; reversed colostomies excluded
Yeung *et al*. (2014) 35	Colostomy including diverting colostomy and colostomy from salvage APR
Meulendijks *et al*. (2014) 26	Colostomy, excluding colostomies reversed during follow‐up
Franco *et al*. (2015) 28	Submission to definitive colostomy excluding preventative colostomies
El‐Hadaad *et al*. (2015) 23	First colostomy
Kichenadasse *et al*. (2007) 57	Sphincter not intact at last follow‐up
	**Progression‐free survival**
Lee *et al*. (2007) 59	First documented relapse in patients who attained complete response
Vordermark *et al*. (2002) 60	Histological evidence of tumour recurrence or death
Milano *et al*. (2005) 61	Persistent disease after treatment, local failure or distant failure
Koerber *et al*. (2014) 25	Progressive disease or death
James *et al*. (2013) 8	Progressive disease, local recurrence, metastases or death from any cause
Matzinger *et al*. (2009) 24	Relapse or death of any cause

APR, abdominoperineal resection.

Progression‐free survival was reported in 11 studies and defined in eight of these using seven different definitions (Table**** 4). The two studies that specified ‘progressive disease’ as an event for progression‐free survival 8, 25 cited the RECIST 37 for defining progressive disease.

### Delivery of care

The only delivery of care domain outcome was compliance with planned treatment, reported in 11 (12%) follow‐up studies and one cross‐sectional study with an accompanying definition in six studies.

### Toxicity

**Figure 2:**
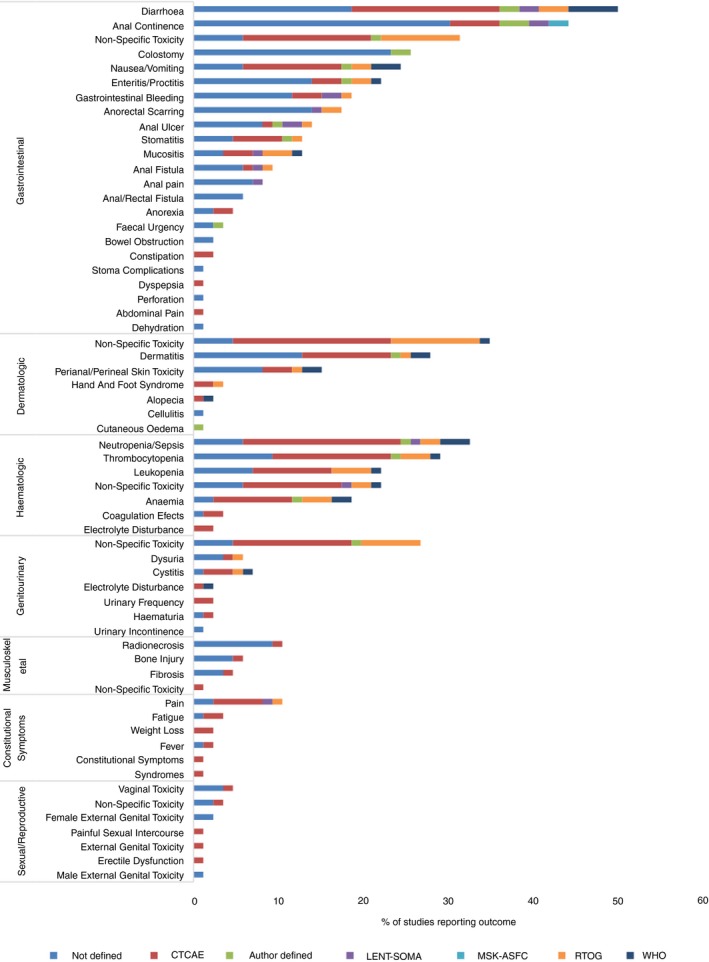
Percentage of studies reporting toxicity outcomes and instruments used. CTCAE, Common Terminology Criteria for Adverse Events; LENT‐SOMA, Late Effects Normal Tissues Subjective, Objective, Management, Analytic Scales; MSK‐ASFC, Memorial Sloan Kettering Anal Sphincter Functioning Criteria; RTOG, Radiation Therapy Oncology Group radiation morbidity scoring schema; WHO, World Health Organization acute toxicity scoring system.

A toxicity outcome was reported in 76 follow‐up studies and six cross‐sectional studies (Fig.**** 2). Gastrointestinal toxicity was the most commonly reported toxicity domain, with diarrhoea the most commonly reported standardized outcome term, reported in 42 (44%) of the 95 studies. Dermatologic toxicity was reported in 57 (60%) studies but in 25 (43%) of these, no further detail was provided. Over one‐quarter (25 of 95) of studies reported nonspecified toxicity, providing no information on the body system(s) involved.

Forty‐nine (51%) studies reported at least one toxicity outcome without an accompanying description of how the toxicity was defined or measured. The most commonly cited criteria for defining and grading toxicity was the Common Terminology Criteria for Adverse Events (CTCAE), reported in 26 studies. Nine studies used author‐defined criteria for defining and grading toxicity.

Anal continence, incorporating incontinence to faeces or flatus, was reported in 39 (41%) studies. Forty‐six unique outcome terms describing anal continence were identified. No single outcome term was reported in more than six studies, and 39 outcomes were reported in a single study only. Twenty‐six (67%) of the 39 studies reporting anal continence provided no accompanying definition. The most frequently reported criteria for defining and grading anal continence was the CTCAE (five studies) (Fig.**** 2). Faecal urgency was reported in three (3%) studies with an accompanying definition in only one study.

### Life Impact

The composite life impact outcome of HRQoL was reported in five (6%) of 86 follow‐up studies and in all nine (100%) cross‐sectional studies. The most frequently used tool for assessing HRQoL was the EORTC‐C30, in nine studies. The most frequently used combination of HRQoL instruments was EORTC‐C30 with EORTC‐38, in five studies. Outcomes extracted from the HRQoL instruments are shown in Table S5. Extracted outcomes include patient‐reported physiological outcomes, for example gastrointestinal and constitutional symptoms, as well as functional life impact outcomes, such as social and emotional function. No measurement instrument included outcomes related to skin toxicity.

## Discussion

Using a systematic approach, we identified 567 unique outcome terms reported in studies evaluating radiotherapy or chemoradiotherapy in patients with ASCC. No single outcome was reported in all trials and over half of outcomes were reported in five studies or fewer. Only half of outcomes were accompanied by any definition and there was wide variation in definitions of disease‐free, colostomy‐free, and progression‐free survival, common secondary outcomes in trials. Toxicity and life impact domains, representing outcomes likely to be important to patients, were poorly represented. Chemoradiotherapy is principally advocated to preserve anal sphincter function, yet outcomes describing adequacy of anorectal function, such as anal continence and faecal urgency, were infrequently reported and inconsistently defined.

The level of observed outcome heterogeneity may be due to a number of factors. Traditionally, overall survival has been considered as the gold standard outcome for cancer clinical trials 38, 39 and is the most commonly reported survival outcome in cancer randomized trials 40. However, the effectiveness of modern cancer interventions means that adequately powering studies for this outcome requires large numbers of study participants and a long period of follow‐up 41. Trialists have adopted surrogate survival outcomes that can be assessed earlier; however, few surrogate survival outcomes have been shown to correlate accurately with overall survival and no global consensus has been reached on the optimal surrogate outcome 42. As a result, a range of survival and composite survival outcomes have been used, further confounded by the use of varying definitions for the same outcome.

Another factor that may explain the number of outcomes observed in this review is the increasing inclusion of HRQoL instruments and patient‐reported outcomes measures (PROMs) in trials. Including patients’ perspectives about their health in the evaluation of treatment interventions is a key component of the patient‐centred model of care 43. However, until 2017 there was no measurement instrument for HRQoL validated for use in patients receiving treatment for anal cancer 44. The use of numerous different HRQoL instruments in differing combinations highlighted in this review is probably a reflection of the inadequacy of the existing instruments in representing the unique issues experienced by patients undergoing treatment for anal cancer 45.

To our knowledge, this is the first systematic review of outcome measures in patients with anal cancer. A recent narrative review from Glynne‐Jones *et al*. 13, critiquing six Phase III randomized trials of interventions for ASCC, concluded that the quality of outcome reporting in those trials was inconsistent. The authors broadly divided discussion on outcomes into ‘time‐to‐event endpoints’ (survival and composite survival outcomes) and ‘non‐time‐to‐event endpoints’ (including tumour response, adverse events, acute toxicity, toxic deaths, late effects, tolerability compliance and patient‐reported outcomes). Their approach may be too simplistic as several of these ‘non‐time to event endpoints’ might vary with time, and patient‐reported outcomes can include outcomes within other categories, for example, acute toxicity. Our review included a wider range of study types and outcomes. We categorized identified outcomes into five broad domains in a process involving discussion amongst our expert study advisory group which, critically, includes a patient representative. These domains map to the universal outcome taxonomy proposed by the COMET group 20. Through detailed, multilevel categorization of the domains of toxicity (for example, according to anatomic site) and life impact, we ensured inclusion of clinically relevant, but infrequently reported, outcomes. For example, we identified outcomes relating to stoma complications, urinary incontinence and erectile dysfunction, each reported in a single study. Less than half of the studies reported any measure of anal continence, and only three studies reported faecal urgency. Yet, in our clinical practice, these symptoms represent a proportionately common set of late effects experienced by anal cancer survivors. Glynne‐Jones *et al*. 13 recognize anal function as likely to be important to patients and propose anal‐dysfunction free survival as a new composite outcome. However, research exploring the priorities of patients with anal cancer is scarce, and further evidence is needed to ensure patient priorities are accurately represented.

This study has some limitations. The systematic review was limited to English language articles; therefore, there may be reduced generalizability by omission of any outcomes reported exclusively in the non‐English literature. However, we identified nearly 100 standardized outcome terms and therefore it seems unlikely that we are missing many outcomes. We plan to mitigate against this potential gap by inviting patient and health‐care professional (HCP) participants in subsequent stages of the COS development process to suggest additional outcomes for consideration. We limited our search to studies evaluating radiotherapy and chemoradiotherapy in the nonmetastatic setting, corresponding to the specific scope of the COS we are developing. The two other main clinical settings on the anal cancer pathway are radical salvage surgery after local relapse and treatment of metastatic disease. There may be specific outcomes relevant to these settings (for example, surgical complications), and these require further research.

Our findings of (i) significant heterogeneity of outcomes across the literature, (ii) inconsistent or absent outcome definition and (iii) neglected reporting of specific toxicity and QoL outcomes, clearly illustrate the need for an agreed, standardized core outcome set to be measured and reported on in all future trials in this field. The COMET initiative recommends that COS development utilizes rigorous consensus methods which involve all stakeholders, including patients. Having performed this systematic review of the literature we are developing a COS for treatment and trials in anal cancer, utilizing a recognized stepwise process of information gathering followed by consensus techniques 46. We will combine the 86 standardized outcome terms identified in this review with outcomes identified through a series of semi‐structured interviews with patients, to produce an exhaustive list of outcomes. This list will be used to create an internationally run Delphi survey involving patients and HCPs. The results of the Delphi process will be discussed at a face‐to‐face consensus meeting where the final COS will be agreed. Additional work will then be required to determine how each of these outcomes should be defined and measured (a core outcome measurement instrument set). Data gathered for this systematic review will facilitate the first step in agreeing the ‘how’, through identification of existing measurement instruments.

## Funding

This paper presents independent research funded by the National Institute for Health Research (NIHR) under its Research for Patient Benefit (RfPB) Programme (Grant Reference Number PB‐PG‐1013‐32064). The views expressed are those of the authors and not necessarily those of the NHS, the NIHR or the Department of Health.

SNV is supported by The Farr Institute @ HeRC. The Farr Institute @ HeRC is supported by a 10‐funder consortium: Arthritis Research UK, the British Heart Foundation, Cancer Research UK, the Economic and Social Research Council, the Engineering and Physical Sciences Research Council, the Medical Research Council, the National Institute of Health Research, the National Institute for Social Care and Health Research (Welsh Assembly Government), the Chief Scientist Office (Scottish Government Health Directorates), and the Wellcome Trust, (MRC Grant No: MR/K006665/1).

## Supporting information


**Table S1.** List of verbatim outcomes, ‘standardised outcome terms’ and domains.
**Table S2.** Summaries for study characteristics.
**Table S3.** Standardised outcome terms identified by categories of study numbers.
**Table S4.** Criteria used to define treatment response.
**Table S5.** Outcomes extracted from Quality of Life Measurement Instruments.
