# Supplementary material for: Systematic review of outcome measures following chemoradiotherapy for the treatment of anal cancer (CORMAC)
Source: Colorectal Dis. 2018 Apr 17;20(5):371–82. doi: 10.1111/codi.14103 (PMC5969105; doi:10.1111/codi.14103)
Supplement: Supplementary file 1 — Table S1. List of verbatim outcomes, ‘standardised outcome terms’ and domains. Table S2. Summaries for study characteristics. Table S3. Standardised outcome terms identified by categories of study numbers. Table S4. Criteria used to define treatment response. Table S5. Outcomes extracted from Quality of Life Measurement Instruments. [file CODI-20-371-s001.doc]

# **Supplementary material**

# Systematic review of outcome measures following chemo-radiotherapy for the treatment of anal cancer (CORMAC)

**Ms Rebecca Fish^1,2^; Dr Caroline Sanders^3^; Mr Neil Ryan^4^; Dr Sabine Van der Veer^,5,6^; Professor Andrew G. Renehan^1,2*^ ; Professor Paula R. Williamson^7,*^**

*Joint senior authors

1. Division of Cancer Sciences, School of Medical Sciences, Faculty of Biology, Medicine and Health, University of Manchester, Vaughan House, Portsmouth Street, Manchester, M13 9GB.
2. Peritoneal and Colorectal Oncology Centre, Christie NHS Foundation Trust, 550 Wilmslow Road, Manchester, M20 4BX.
3. Centre for Primary Care, University of Manchester, Williamson Building, 6^th^ Floor, Suite 3, Manchester, M13 9PL.
4. Division of Cancer Sciences, School of Medical Sciences, Faculty of Biology, Medicine and Health Fifth Floor - Research, St Mary’s Hospital, University of Manchester, Oxford Road, Manchester M13 9WL, UK.
5. Centre for Health Informatics, Informatics, Imaging and Data Science, School of Health Sciences, Faculty of Biology, Medicine and Health, University of Manchester, Vaughan House, Portsmouth Street, Manchester, M13 9GB.
6. Farr Institute of Health Informatics Research, Health eResearch Centre, University of Manchester, Manchester, UK
7. MRC North West Hub for Trials Methodology Research, Department of Biostatistics, Block F Waterhouse Building, University of Liverpool, 1-5 Brownlow Street, Liverpool, L69 3GL.

Correspondence to:

Dr Rebecca Fish

Clinical Research Fellow

Division of Cancer Sciences, School of Medical Sciences

Faculty of Biology, Medicine and Health

University of Manchester

Vaughan House, Portsmouth Street

Manchester, M13 9GB.

E-mail: [rebecca.fish-2@manchester.ac.uk](mailto:andrew.renehan@ics.manchester.ac.uk)

Tables 5; References 25

**Table S1: List of verbatim outcomes, ‘standardised outcome terms’ and domains**

| **Outcome domain** | **Standardised outcome term** | **Verbatim outcomes** |
| --- | --- | --- |
| **Survival** | Disease specific survival | Cancer specific survival |
|  |  | Cause specific survival |
|  |  | Death from anal cancer |
|  |  | Death of disease |
|  |  | Died of disease |
|  |  | Disease specific survival |
|  |  | Tumour specific survival |
|  | Overall survival | Absolute survival |
|  |  | Actuarial survival |
|  |  | Crude survival |
|  |  | Death |
|  |  | Died of intercurrent disease |
|  |  | Dying |
|  |  | Overall survival |
|  |  | Survival |
| **Survival composite** | Colostomy free survival | Colostomy free survival |
|  |  | Disease free survival |
|  | Complication free survival | Severe complication free survival |
|  | Disease free survival | Alive no evidence of disease |
|  |  | Cause specific survival |
|  |  | Disease free |
|  |  | Disease free survival |
|  |  | No evidence of disease survival |
|  |  | Primary relapse free survival |
|  |  | Tumour free survival |
|  | Event free survival | Event free survival |
|  | Inguinal relapse free survival | Inguinal relapse free survival |
|  | Local recurrence free survival | Local recurrence free survival |
|  |  | Local relapse free survival |
|  | Metastases free survival | Distant metastasis free survival |
|  | Progression free survival | Progression free survival |
|  |  | Recurrence free survival |
|  |  | Relapse free survival |
|  | Relapse free survival | Relapse free survival |
| **Disease activity** | Disease progression | Disease progression |
|  |  | Failure of primary therapy |
|  |  | Failure of treatment |
|  |  | Freedom from recurrence |
|  |  | Freedom from recurrent tumour |
|  |  | Pattern of failure |
|  |  | Pattern of relapse |
|  |  | Patterns of failure |
|  |  | Persistent disease |
|  |  | Recurrence |
|  |  | Recurrent disease |
|  |  | Relapse |
|  |  | Relapse after complete response |
|  |  | Relapse free interval |
|  |  | Residual or recurrent tumour |
|  |  | Site of recurrence |
|  |  | Unsalvagable relapse |
|  | Local disease activity | Anal margin recurrence |
|  |  | Complete local control |
|  |  | freedom from local recurrence |
|  |  | Isolated local failure |
|  |  | Local and inguinal recurrence |
|  |  | Local control |
|  |  | Local control with anal function |
|  |  | Local control with preserved sphincter |
|  |  | Local failure |
|  |  | Local progressive tumour |
|  |  | Local recurrence |
|  |  | Local relapse |
|  |  | Local treatment failure |
|  |  | Local tumour control |
|  |  | Local tumour relapse |
|  |  | Persistent local disease |
|  |  | Primary anal tumour control |
|  |  | Primary relapse free |
|  |  | Primary tumour control |
|  |  | Vaginal local recurrence |
|  | Locoregional disease activity | In field recurrence |
|  |  | Local regional control |
|  |  | Local/locoregional tumor relapse |
|  |  | Locoregional control |
|  |  | Locoregional disease free control |
|  |  | Locoregional failure |
|  |  | Loco-regional failure |
|  |  | Locoregional recurrence |
|  |  | Loco-regional recurrence |
|  |  | Locoregional relapse |
|  |  | Locoregional treatment failure |
|  |  | Locoregional tumour recurrence |
|  |  | Pelvic recurrence |
|  | Metastatic disease activity | Distant metastases |
|  |  | Distant control |
|  |  | Distant failure |
|  |  | Distant recurrence |
|  |  | Distant relapse |
|  |  | Distant relapses |
|  |  | Distant treatment failure |
|  |  | Distant tumor relapse |
|  |  | Extrapelvic metastases |
|  |  | Freedom from metastases |
|  |  | Liver metastasis |
|  |  | Metastases |
|  |  | Metastasis free survival |
|  |  | Metastatic failure |
|  |  | Recurrence in the liver |
|  |  | Systematic failure |
|  |  | Systemic failure |
|  |  | Systemic relapse |
|  | Nodal disease activity | Iliac node recurrence |
|  |  | Inguinal failure |
|  |  | Inguinal lymph nodal control |
|  |  | Inguinal lymph node failure |
|  |  | Inguinal nodal failure |
|  |  | Inguinal node failure |
|  |  | Inguinal node recurrence |
|  |  | Inguinal progression or relapse |
|  |  | Inguinal recurrence |
|  |  | Local inguinal nodal relapse |
|  |  | Local lymph nodal relapse |
|  |  | Perirectal node recurrence |
|  |  | Regional lymph node failure |
|  |  | Regional node control |
|  |  | Regional nodes controlled |
|  | Regional disease activity | Regional control |
|  |  | Regional failure |
|  |  | Regional recurrence |
|  |  | Regional recurrences |
|  |  | Regional relapse |
|  |  | Regional tumour control |
|  | Treatment response | Clinical complete response |
|  |  | Clinical remission |
|  |  | Clinical response |
|  |  | Complete clinical response |
|  |  | Complete locoregional response |
|  |  | Complete remission |
|  |  | Complete response |
|  |  | Complete tumour regression |
|  |  | Complete tumour response |
|  |  | Freedom from residual tumour |
|  |  | Negative post induction biopsy |
|  |  | Objective tumour response |
|  |  | Pathologically confirmed response |
|  |  | Residual tumour |
|  |  | Response |
|  |  | Response to treatment |
|  |  | Treatment response |
|  |  | tumour regression |
|  |  | Tumour remission |
|  |  | Tumour response |
| **Life impact** | Cognitive functioning | Concentration |
|  | Health related quality of life | Global quality of life |
|  |  | Quality of life |
|  | Physical functioning | Physical functioning |
|  | psychological functioning | Feeling alone |
|  |  | Normality |
|  |  | Stability |
|  |  | Stress |
|  | Role | Duration of sick leave |
|  |  | Return to work |
|  | Social functioning | Changes in interaction with family and friends |
|  |  | Embarrassment |
|  |  | Feeling mistrusted and devalued |
|  |  | Finding a partner |
|  |  | Imposed discrete/modest behaviour |
|  |  | Relationships |
|  |  | Social functioning |
|  |  | Social stigma |
|  |  | Stigma |
| **Delivery of care** | Compliance | Adherence to protocol |
|  |  | Chemotherapy adherence |
|  |  | Compliance |
|  |  | Dose reduction |
|  |  | Interruption of treatment due to toxicity |
|  |  | Overall compliance |
|  |  | Overall treatment time |
|  |  | Radiotherapy adherence |
|  |  | Treated according to guidelines |
|  |  | Treatment compliance |
|  |  | Treatment interruption |
|  |  | Unscheduled treatment break |
|  | Delivery of care | Patient preference regarding treatment modality |
| **Gastrointestinal toxicity** | Abdominal pain | Colic pain |
|  | Anal continence | Alive with functional anus |
|  |  | Anal continence |
|  |  | Anal discharge |
|  |  | Anal function |
|  |  | Anal functioning |
|  |  | Anal incompetence |
|  |  | Anal incontinence |
|  |  | Anal preservation |
|  |  | Anal sphincter preservation |
|  |  | Anorectal excision |
|  |  | Anorectal function |
|  |  | Anorectal manometry |
|  |  | Continence |
|  |  | Faecal accident |
|  |  | Faecal incontinence |
|  |  | Functional anal sphincter |
|  |  | Functional anorectal sphincter |
|  |  | Functional sphincter |
|  |  | Functioning anal canal |
|  |  | Impaired anorectal function |
|  |  | Incontinence |
|  |  | Incontinence for gas |
|  |  | Insufficient sphincter function |
|  |  | Intact anal function |
|  |  | Light dysfunction |
|  |  | Light incontinence |
|  |  | Manometric sphincter length |
|  |  | Maximum squeeze pressure |
|  |  | Metabolic/laboratory |
|  |  | Moderate dysfunction |
|  |  | Normal anal function |
|  |  | Partially incontinent |
|  |  | permanent loss of anal sphincter function |
|  |  | Preservation of normal sphincter function |
|  |  | Preservation of sphincter function |
|  |  | Preserved sphincter |
|  |  | Rectal compliance |
|  |  | Relaxation of internal sphincter |
|  |  | Residual sphincteric incontinence |
|  |  | resting pressure |
|  |  | Retained anal sphincter |
|  |  | Salvage surgery |
|  |  | Satisfaction with anorectal function |
|  |  | Slight incontinence |
|  |  | Soiling |
|  |  | Sphincter damage |
|  |  | Sphincter function |
|  |  | Sphincter preservation |
|  |  | Stool incontinence |
|  | Anal fistula | Anal fistula |
|  |  | Fistula |
|  |  | Rectovaginal fistulae |
|  |  | Ulceration / fistula |
|  | Anal pain | Anal pain |
|  |  | Ano genital pain |
|  |  | Ano-genital pain |
|  |  | Perianal pain |
|  |  | Rectal discomfort |
|  | Anal ulcer | anal ulcer |
|  |  | Anal ulceration |
|  |  | Anal ulcers |
|  |  | Anorectal ulcer |
|  |  | Chronic ulceration |
|  |  | Post implant ulceration |
|  |  | Ulcer |
|  |  | Ulceration |
|  |  | Ulceration in the anal canal |
|  |  | Ulcers |
|  | Anal/rectal fistula | Rectovaginal fistula |
|  |  | Recto-vaginal fistula |
|  | Anorectal scarring | Anal fibrosis |
|  |  | Anal stenosis |
|  |  | Anal stricture |
|  |  | Ano-rectal fibrosis |
|  |  | Fibrosis in the sphincter apparatus |
|  |  | Rectal fibrosis |
|  |  | Rectal stenosis |
|  |  | Rectal stenosis with faecal incontinence |
|  |  | Stenosis |
|  |  | Stricture |
|  | Anorexia | Anorexia |
|  |  | Poor appetite |
|  | Bowel obstruction | Intestinal obstruction |
|  |  | Small bowel obstruction |
|  | Colostomy | Colostomy |
|  |  | Colostomy failure |
|  |  | Colostomy free interval |
|  |  | Colostomy rate |
|  |  | Sigmoidostomy |
|  |  | Time to colostomy |
|  | Constipation | Constipation |
|  | Dehydration | Dehydration |
|  | Diarrhoea | Acute diarrhoea |
|  |  | Chronic diarrhoea |
|  |  | Diarrhoea |
|  |  | Increased frequency of bowel movements |
|  |  | Looser stools than normal |
|  | Dyspepsia | Dyspepsia |
|  | Enteritis/proctitis | Anorectal mucosal angiodysplasia |
|  |  | Chronic hemorrhagic proctitis |
|  |  | Chronic proctitis |
|  |  | Colitis |
|  |  | Enteritis |
|  |  | Enterocolitis |
|  |  | Necrosis of the rectum |
|  |  | Proctitis |
|  |  | Radiation enteritis |
|  |  | Radiation proctitis |
|  |  | Rectocolitis |
|  | Faecal urgency | Faecal urgency |
|  |  | Stool urgency |
|  |  | Urgency |
|  |  | Urgency for defaecation |
|  | Gastrointestinal bleeding | Anal haemorrhage |
|  |  | Anorectal bleeding |
|  |  | Bleeding |
|  |  | Gastroinstestinal haemorraghe |
|  |  | Haemoarrhage |
|  |  | Haemorrhage |
|  |  | Intermittent benign bleeding |
|  |  | Intestinal bleeding |
|  |  | Lower gastrointestinal bleeding |
|  |  | Occasional fresh bleeding (in the stool) |
|  |  | Rectal bleeding |
|  | Mucositis | Anal mucositis |
|  |  | Mucosal ulceration |
|  |  | Mucositis |
|  |  | Oral mucositis |
|  | Nausea/vomiting | Nausea |
|  |  | Nausea / vomiting |
|  |  | Nausea and vomiting |
|  |  | Vomiting |
|  | Non-specific toxicity | Acute gastrointestinal toxicity |
|  |  | Acute lower gastrointestinal toxicity |
|  |  | Acute lower intestinal toxicity |
|  |  | Acute upper gastrointestinal toxicity |
|  |  | Bowel complications |
|  |  | Bowel dysfunction |
|  |  | Bowel function |
|  |  | Bowel reactions |
|  |  | Chronic gastrointestinal toxicity |
|  |  | Early morbidity gastrointestinal |
|  |  | Gastrointestinal |
|  |  | Gastrointestinal bleeding |
|  |  | Gastrointestinal toxicity |
|  |  | GI toxicity |
|  |  | Haematologic toxicity |
|  |  | Intestinal problems |
|  |  | Late gastrointestinal toxicity |
|  |  | Late morbidity gastrointestinal |
|  |  | Rectal / intestinal toxicity |
|  |  | Rectal toxicity |
|  |  | Side effects related to the anus-rectum |
|  |  | Small / large intestine |
|  |  | Small/large intestine |
|  | Pelvic organ prolapse | Prolapse |
|  | Perforation | perforation |
|  | Stoma complications | Stoma prolapse |
|  | Stomatitis | Acute stomatitis |
|  |  | Stomatitis |
|  |  | Stomatitis/mucositis |
| **Dermatologic toxicity** | Alopecia | Alopecia |
|  |  | Hair loss |
|  | Cellulitis | Purulent cellulitis |
|  | Cutaneous oedema | Oedema |
|  | Dermatitis | Dermatitis |
|  |  | Desquamating dermatitis |
|  |  | Erythema |
|  |  | Hyperpigmentation |
|  |  | Inguinal dermatitis |
|  |  | Moist desquamation |
|  |  | Moist desquamation in perianal area |
|  |  | Perineal moist desquamation |
|  |  | Perineal reaction |
|  |  | Radiation dermatitis |
|  |  | Radiation induced dermatitis |
|  |  | Radiodermatitis |
|  |  | Rash |
|  |  | Skin atrophy |
|  |  | Skin reaction |
|  |  | Skin ulceration |
|  |  | Telangectasia |
|  | Hand and foot syndrome | Hand and feet syndrome |
|  |  | Hand and foot syndrome |
|  |  | Palmar/plantar |
|  | Non-specific toxicity | Acute dermatologic toxicity |
|  |  | Acute skin toxicity |
|  |  | Burning to skin |
|  |  | Chronic skin toxicity |
|  |  | Cutaneous |
|  |  | cutaneous toxicity |
|  |  | Dermatologic toxicity |
|  |  | Dermatology/skin |
|  |  | Early morbidity skin |
|  |  | Late morbidity skin |
|  |  | Late skin toxicity |
|  |  | Skin |
|  |  | Skin reaction |
|  |  | Skin reactions |
|  |  | Skin toxicity |
|  |  | Skin/mucosal reactions |
|  |  | Subcutaneous tissue |
|  | Perianal/perineal skin toxicity | Atrophy of perianal skin |
|  |  | Cutaneous perianal necrosis and ulceration |
|  |  | Genital dermatitis |
|  |  | Peri-anal cutaneous ulceration |
|  |  | Perianal desquamation |
|  |  | Perianal radiation dermatitis |
|  |  | Perianal ulceration |
|  |  | Perineal |
|  |  | Perineal dermatitis |
|  |  | Perineal skin reaction |
|  |  | Perineal skin toxicity |
|  |  | Perineal skin ulceration |
|  |  | Severe perineal reaction |
| **Haematologic toxicity** | Anaemia | Anaemia |
|  |  | Thrombocytopenia |
|  | Coagulation effects | Arterial thrombosis |
|  |  | Coagulation |
|  |  | Thrombosis / Embolism |
|  | Electrolyte disturbance | Hyperkalaemia |
|  |  | Hyponatraemia |
|  |  | Magnesium wasting syndrome |
|  | Leukopenia | Leucopenia |
|  |  | Leukocytopenia |
|  |  | Leukopenia |
|  |  | WBC toxicity |
|  |  | White blood cell count |
|  | Neutropenia/sepsis | Febrile neutropenia |
|  |  | Febrile neutropneia requiring hospitalisation |
|  |  | Infection |
|  |  | Infection / febrile neutropenia |
|  |  | Infection without neutropenia |
|  |  | Infection/febrile neutropenia |
|  |  | Neutropenia |
|  |  | Neutropenia/granulocytopenia |
|  |  | Sepsis |
|  |  | Septicaemia |
|  |  | Wound infection |
|  | Non-specific toxicity | Acute haematologic toxicity |
|  |  | Blood/bone marrow |
|  |  | Early morbidity haematological |
|  |  | Haematologic toxicity |
|  | Thrombocytopenia | Aplasia |
|  |  | Early morbidity platelets |
|  |  | Leukopenia and thrombocytopenia |
|  |  | Leukopenia/thrombocytopenia |
|  |  | Platelet count |
|  |  | Platelet counts <100,000/mm3 |
|  |  | Platelets |
|  |  | Thrombocytopaenia |
| **Musculoskeletal toxicity** | Bone injury | Femoral fracture |
|  |  | femoral neck fracture |
|  |  | Hip/pelvis fracture |
|  |  | Insufficiency fracture |
|  |  | Pelvic failure |
|  | Fibrosis | Fibrosis |
|  |  | Inguinal fibrosis |
|  |  | Retroperitoneal fibrosis |
|  |  | Severe fibrosis |
|  | Non-specific toxicity | Acute osseous toxicity |
|  | Radionecrosis | Anal and perineal necrosis |
|  |  | anal canal necrosis |
|  |  | Necrosis |
|  |  | Painful necrosis |
|  |  | Radionecrosis |
|  |  | Skin necrosis |
|  |  | Soft tissue necrosis |
|  |  | Ulceration/necrosis |
| **Urinary toxicity** | Cystitis | Chronic cystitis |
|  |  | Cystitis |
|  |  | Haemorrhagic cystitis |
|  | Dysuria | Dysuria |
|  |  | Urethral stricture |
|  | Electrolyte disturbance | Acute renal insufficiency |
|  |  | Elevated creatinine value |
|  | Haematuria | Haematuria |
|  | Non-specific toxicity | Acute bladder toxicity |
|  |  | Acute genitourinary toxicity |
|  |  | Bladder |
|  |  | Bladder toxicity |
|  |  | Early morbidity genitourinary |
|  |  | Genitourinary |
|  |  | Genitourinary toxicity |
|  |  | Late genitourinary toxicity |
|  |  | Late morbidity genitourinary |
|  |  | Renal |
|  |  | Renal toxicity |
|  |  | Renal/genitourinary |
|  |  | Side effects related to the urinary tract |
|  |  | Urinary |
|  |  | Urinary symptoms |
|  |  | Urinary tract complications |
|  | Urinary frequency | Urinary frequency |
|  | Urinary incontinence | Urinary incontinence |
|  | Urinary urgency | Urinary urgency |
| **Sexual and reproductive toxicity** | Erectile dysfunction | Erectile dysfunction |
|  |  | Erective capability |
|  |  | Impotence |
|  | External genital toxicity | Ano genital mucosa damage |
|  |  | Ano genital skin damage |
|  |  | Atrophy to perineal skin and external genitalia |
|  |  | Chronic genital mucosal toxicity |
|  | Female external genital toxicity | Perianal or vulvar skin reaction |
|  |  | Vulval oedema |
|  | Libido | Decreased libido |
|  | Male external genital toxicity | Penile oedema |
|  | Non-specific toxicity | Sexual activity |
|  |  | Sexual dysfunction |
|  |  | Sexual problems |
|  |  | Sexual/reproductive function |
|  | Painful sexual intercourse | Dyspareunia |
|  | Sexual pleasure | Intimacy |
|  | Vaginal toxicity | Atrophy of vaginal mucosa |
|  |  | Colpitis |
|  |  | Vaginal discharge |
|  |  | Vaginal dryness |
|  |  | Vaginal fibrosis |
|  |  | Vaginal stenosis |
|  |  | Vaginal stricture |
| **Constitutional symptoms** | Constitutional symptoms | Constitutional symptoms |
|  | Fatigue | Fatigue |
|  |  | General fatigue |
|  |  | Lethargy |
|  | Fever | Fever |
|  | Pain | Chronic pain |
|  |  | Pain |
|  |  | Thoracic pain |
|  | Syndromes | Syndromes |
|  | Weight loss | Weight loss |
| **Cardiovascular toxicity** | Cardiovascular | Arrhythmias |
|  |  | Chest pain |
|  |  | Phlebitis |
|  | Non-specific toxicity | Cardiac |
|  |  | Cardiovascular (general) |
|  |  | Vascular |
| **Endocrine toxicity** | Non-specific toxicity | Endocrine |
| **Hepatic toxicity** | Hepatic toxicity | Gamma-glutamyltransferase |
|  | Non-specific toxicity | Hepatic |
| **Immunologic toxicity** | Allergy | Allergy/Immunology |
| **Lymphatic toxicity** | Lymphatic | Leg lymphedema |
|  |  | Leg oedema |
|  |  | Lower extremity oedema |
|  |  | Lymphatics |
| **Neurologic toxicity** | Nneurologic toxicity | Auditory/hearing |
|  |  | Encephalopathy |
|  |  | Neurotoxicity |
|  |  | Occular/visual |
|  | Non-specific toxicity | Neurologic |
|  |  | Neurological |
|  |  | Neurological toxicity |
|  |  | Neurology |
|  | Perianal numbness | Perinanal numbness |
| **Respiratory toxicity** | Non-specific toxicity | Pulmonary |
|  | Respiratory | Dyspnoea |
| **Second malignancy** | Second malignancy | Second malignancy |
|  |  | Second primary failure |
| **Non-specific toxicity** | Non-specific toxicity | Acute adverse effects |
|  |  | Acute chemotherapy related toxicity |
|  |  | Acute non-haematological toxicity |
|  |  | Acute radiation a related toxicity |
|  |  | Acute toxicity |
|  |  | Adverse side effects of treatment |
|  |  | Chemotherapy toxicity |
|  |  | Complications |
|  |  | Complications of treatment |
|  |  | Fatal late toxicity |
|  |  | Hospitalisation |
|  |  | late adverse effects |
|  |  | Late complications |
|  |  | Late damage |
|  |  | Late morbidity |
|  |  | Late toxicity |
|  |  | Major morbidity |
|  |  | Minor morbidity |
|  |  | Other |
|  |  | Other non-haematologic toxicity |
|  |  | Radiotherapy toxicity |
|  |  | Severe complications |
|  |  | Severe toxicity |
|  |  | Severe toxicity free interval |
|  |  | Toxicity free interval |
|  |  | Toxicity of radiotherapy |
|  |  | Treatment toxicity |

**Table S2:** Summaries for study characteristics

| Characteristic |  | N (total 101) |
| --- | --- | --- |
| Year of publication | 2010-2016 | 37 |
|  | 2000-2009 | 29 |
|  | 1990-1999 | 31 |
|  | 1980-1989 | 4 |
| Type of study | Randomised trial | 11 |
|  | Non-randomised trial | 6 |
|  | Prospective cohort | 16 |
|  | Retrospective cohort | 53 |
|  | Cross sectional | 9 |
|  | Qualitative study | 2* |
|  | Systematic Review | 4* |
| Radiotherapy details | Radiotherapy with or without concurrent chemotherapy | 32 |
|  | Radiotherapy with concurrent chemotherapy | 52 |
|  | IMRT with concurrent chemotherapy | 13 |
|  | Radiotherapy alone | 5 |

IMRT = Intensity modulated radiotherapy

* Outcomes extracted from these studies were included in the outcome long-list for COS development but excluded from the descriptive analysis in this paper

Table S3: Standardised outcome terms identified by categories of study numbers

| 1 study | 2-4 studies | 5-9 studies | 10-19 studies | 20 or more studies |
| --- | --- | --- | --- | --- |
| *(n=21 standardised outcome terms)* | *(n=22 standardised outcome terms)* | *(n=10 standardised outcome terms)* | *(n=14 standardised outcome terms)* | (n=16 standardised outcome terms) |
| | Abdominal pain | | --- | | Allergy | | Cellulitis | | Complication free survival | | Constitutional symptoms | | Cutaneous oedema | | Dehydration | | Dyspepsia | | Erectile dysfunction | | External genital toxicity NOS | | Hepatic toxicity | | Inguinal relapse free survival | | Male external genital toxicity | | Painful sexual intercourse | | Perforation | | Perianal numbness | | Relapse free survival | | Respiratory | | Stoma complications | | Syndromes* | | Urinary incontinence | | | Anorexia | | --- | | Electrolyte disturbance | | Event free survival | | Fibrosis | | Local recurrence free survival | | Metastases free survival | | Vaginal toxicity | | Cardiovascular toxicity | | Coagulation effects | | Faecal urgency | | Fatigue | | Hand and foot syndrome | | Alopecia | | Bowel obstruction | | Constipation | | Female external genital toxicity | | Fever | | Haematuria | | Neurologic toxicity | | Second malignancy | | Urinary frequency | | Weight loss | | | Pain | | --- | | Radionecrosis | | Anal fistula | | Anal pain | | Anal/rectal fistula | | Bone injury | | Cystitis | | Dysuria | | Health related quality of life | | Lymphatic toxicity | | | Disease progression | | --- | | Leukopenia | | Enteritis/proctitis | | Nodal disease activity | | Anaemia | | Gastrointestinal bleeding | | Anorectal scarring | | Perianal/perineal skin toxicity | | Anal ulcer | | Compliance | | Progression free survival | | regional disease activity | | Stomatitis | | Mucositis | | | Overall survival | | --- | | Local disease activity | | Non-specific toxicity | | Treatment response | | Diarrhoea | | Metastatic disease activity | | Anal continence | | Colostomy free survival | | Disease free survival | | Locoregional disease activity | | Neutropenia/sepsis | | Thrombocytopenia | | Dermatitis | | Disease specific survival | | Colostomy | | Nausea/vomiting | |

NOS: not otherwise specified. * ‘Syndromes’ was reported as an outcome with no further definition.

# Table S4: Criteria used to define treatment response

| **Study** | **Verbatim outcome term** | **Timing of assessment**  **(after completion of treatment)** | **Modality** | **Level of response** | | | |
| --- | --- | --- | --- | --- | --- | --- | --- |
|  |  |  |  | Complete | Partial | Stable | Progressive |
|  | | | RECIST (1) | | | | |
| Matthews et al. (2) | Response | 3-6 months | Clinically or radiologically | Disappearance of target lesions | At least 40% reduction in sum of diameters of target lesions | Insufficient shrinkage/growth to qualify for partial response or progressive disease | At least 20% increase in sum of diameters of target lesions |
| Leon et al. (3) | Response | 3 months |  |  |  |  |  |
| El-Hadaad et al. (4) | Response | 4 weeks |  |  |  |  |  |
| Matzinger et al. (5) | Response | 8 weeks |  |  |  |  |  |
| Koerber et al. (6) | Response | 3-6 months |  |  |  |  |  |
| James et al. (7) | Response | 26 weeks |  |  |  |  |  |
|  | | | WHO Criteria (8) | | | | |
| Peiffert et al. (9) | Response | 2 months | Clinically, radiologically, biochemically or pathologically | Disappearance of all known disease on 2 observations 4 weeks apart | 50% or more decrease in tumour load/estimated size | Insufficient shrinkage/growth to qualify for partial response or progressive disease | 25% or more increase in size of tumour |
| Crehange et al. (10) | Objective tumour response | 8 weeks |  |  |  |  |  |
|  | | | Other criteria | | | | |
| Meulendijks et al. (11) | Clinical response | 4-6 weeks | Physical examination | Complete resolution of tumour |  |  |  |
| Baretelink et al. (12) | Response | 8 weeks | Physical examination | Not defined |  |  |  |
| Glynne-Jones et al. (13) | Clinical response | 4 weeks | Physical examination and/or CT/MRI scan | Absence of clinically detectable disease |  |  |  |
| Kouloulias et al. (14) | Response | Not specified | Physical examination and CT scan | 100% tumour regression | 50% decrease in tumour volume | 25% decrease in tumour volume | Increase in tumour volume of >25% |
| Edelman et al. (15) | Tumour response | 6 weeks | Physical examination and endoscopy | No tumour detectable |  |  |  |
| Ferrigno et al. (16) | Response | Not specified | Endoscopy and histology | No tumour detectable |  |  |  |
| Ceresoli (17) | Tumour response | Not specified | Histology | Disappearance of neoplastic cells |  |  |  |
| Grabenbauer et al. (18) | Regression | 6 weeks | Histology | Complete tumour regression |  |  |  |
| Flam et al. (19) | Pathological response | 4-6 weeks | Histology | Negative biopsy |  |  |  |
| Salama et al (20) | Clinical response | 6 weeks | Clinically NOS | No residual tumour | Any residual tumour | Less than 30% response |  |
| Lee et al. (21) | Response | 4 weeks | Clinically NOS | Disappearance of the tumour | >50% reduction in the largest tumour diameter |  |  |
| Martenson et al. (22) | Response | 8 weeks | Clinically NOS | Complete disappearance of detectable disease | Decrease of 50% or more in the product of the longest diameter and the greatest perpendicular diameter |  |  |
| Wagner et al. (23) | Response | Not specified | Not specified | Total regression of the tumour | Regression of more than 50% of the initial volume of the tumour |  |  |
| Sandhu (24) | Response | Not specified | Not specified | According to standard practice | Minimal induration with no evidence of frank tumour |  |  |
| Hwang et al. (25) | Response | 30 days | Not specified | No evidence of disease |  |  |  |

NOS: not otherwise specified; RECIST: Response evaluation criteria in solid tumours; WHO: World Health Organisation

# Table S5: Outcomes extracted from Quality of Life Measurement Instruments

| **Outcome domain** | **Outcome subdomain** | **Standardised outcome term** | **Measurement instrument (number of studies using instrument)** | | | | | | |
| --- | --- | --- | --- | --- | --- | --- | --- | --- | --- |
|  |  |  | EORTC CR29 (2) | EORTC CR38 (5) | EORTC QLQ C30 (9) | FACT-C (3) | FACT-G (1) | GIQLI (2) | MOS sexual problems scale (1) |
| **life impact** | cognitive function | concentration |  |  | 1 |  |  |  |  |
|  |  | memory |  |  | 1 |  |  |  |  |
|  | physical function | help with ADLS |  |  | 1 |  |  |  |  |
|  |  | rest in the day |  |  | 1 |  |  |  |  |
|  |  | strenuous activity |  |  | 1 |  |  |  |  |
|  |  | walking |  |  | 2 |  |  |  |  |
|  | psychological function | anxiety | 1 | 1 | 2 | 2 | 2 | 1 |  |
|  |  | acceptance |  |  |  | 1 | 1 |  |  |
|  |  | body image | 4 | 3 |  | 1 |  | 1 |  |
|  |  | coping |  |  |  | 1 | 1 | 1 |  |
|  |  | depression |  |  | 1 |  |  |  |  |
|  |  | embarrassment | 1 |  |  |  |  |  |  |
|  |  | enjoying life |  |  |  | 2 | 2 |  |  |
|  |  | frustration |  |  |  |  |  | 1 |  |
|  |  | hopelessness |  |  |  | 1 | 1 |  |  |
|  |  | irritability |  |  | 1 |  |  |  |  |
|  |  | mortality |  |  |  | 1 | 1 |  |  |
|  |  | quality of life |  |  |  | 1 | 1 |  |  |
|  |  | sadness |  |  |  | 1 | 1 | 1 |  |
|  | Quality of life | overall health |  |  | 1 |  |  |  |  |
|  |  | quality of life |  |  | 1 |  |  | 1 |  |
|  | role function | family life |  |  |  | 1 | 1 |  |  |
|  |  | financial concerns |  |  | 1 |  |  |  |  |
|  |  | work |  |  | 1 | 2 | 2 | 2 |  |
|  | Social function | family life |  |  | 1 | 3 | 3 |  | 4 |
|  |  | partner |  |  |  | 1 | 1 |  | 7 |
|  |  | socialising |  |  | 2 | 2 | 2 | 1 |  |
| **life impact Total** |  |  | **6** | **4** | **18** | **20** | **19** | **9** | **11** |
| **Toxicity/ Physiological** | constitutional symptoms | fatigue |  |  | 2 | 3 | 3 | 4 |  |
|  |  | insomnia |  |  | 1 |  |  |  |  |
|  |  | non-specific toxicity |  |  |  | 1 | 1 | 1 |  |
|  |  | pain |  |  | 2 | 1 | 1 |  |  |
|  |  | sleep disturbance |  |  |  | 1 | 1 | 1 |  |
|  |  | strength |  |  |  |  |  | 1 |  |
|  |  | weakness |  |  | 1 |  |  |  |  |
|  |  | weight loss |  | 1 |  |  |  |  |  |
|  | dermatologic | alopecia | 1 | 1 |  |  |  |  |  |
|  | gastrointestinal | abdominal gurgling |  |  |  |  |  | 1 |  |
|  |  | abdominal pain | 1 | 1 |  |  |  | 1 |  |
|  |  | anal continence | 3 | 2 |  | 1 |  | 2 |  |
|  |  | anogenital pain | 1 | 1 |  |  |  |  |  |
|  |  | anogential pain | 1 |  |  |  |  |  |  |
|  |  | anorexia |  |  | 1 | 1 |  | 2 |  |
|  |  | belching |  | 1 |  |  |  | 1 |  |
|  |  | bloating | 1 | 1 |  | 1 |  | 2 |  |
|  |  | Constipation |  | 1 | 1 |  |  | 1 |  |
|  |  | diarrhoea |  |  | 1 | 1 |  | 1 |  |
|  |  | dietary modification |  |  |  |  |  | 1 |  |
|  |  | Dry mouth | 1 | 1 |  |  |  |  |  |
|  |  | dyspepsia |  |  |  | 1 |  | 2 |  |
|  |  | dysphagia |  |  |  |  |  | 1 |  |
|  |  | faecal frequency | 2 | 2 |  |  |  | 1 |  |
|  |  | faecal urgency |  | 1 |  |  |  | 1 |  |
|  |  | gastrointestinal bleeding | 1 | 1 |  |  |  | 1 |  |
|  |  | nausea/vomiting |  |  | 2 | 1 | 1 | 1 |  |
|  |  | painful defecation |  | 1 |  |  |  |  |  |
|  |  | stoma complications | 8 | 8 |  | 3 |  |  |  |
|  |  | taste | 1 | 1 |  |  |  |  |  |
|  |  | weight loss |  |  |  | 1 |  |  |  |
|  | respiratory | Dyspnoea |  |  | 1 |  |  |  |  |
|  | sexual function | ejaculatory function |  | 1 |  |  |  |  |  |
|  |  | impotence | 1 | 1 |  |  |  |  | 1 |
|  |  | libido | 1 | 1 |  |  |  |  | 1 |
|  |  | orgasmic function |  |  |  |  |  |  | 1 |
|  |  | painful sexual intercourse | 1 | 1 |  |  |  |  |  |
|  |  | sexual function |  | 1 |  | 1 | 1 | 1 |  |
|  |  | Sexual pleasure |  | 1 |  |  |  |  | 2 |
|  |  | vaginal dryness |  | 1 |  |  |  |  |  |
|  | urinary | dysuria | 1 | 1 |  |  |  |  |  |
|  |  | urinary continence | 1 |  |  |  |  |  |  |
|  |  | urinary frequency | 2 | 2 |  |  |  |  |  |
| **Toxicity / Physiological Total** |  |  | 28 | 34 | 12 | 17 | 8 | 27 | 5 |
| **Grand Total** |  |  | 34 | 38 | 30 | 37 | 27 | 36 | 16 |

Numbers in cells show the number of verbatim question items summarised into each standardised outcome term

# References

1. Eisenhauer EA, Therasse P, Bogaerts J, Schwartz LH, Sargent D, Ford R, et al. New response evaluation criteria in solid tumours: revised RECIST guideline (version 1.1). Eur J Cancer. 2009 Jan;45(2):228-47. PubMed PMID: 19097774.

2. Matthews JH, Burmeister BH, Borg M, Capp AL, Joseph D, Thompson KM, et al. T1-2 anal carcinoma requires elective inguinal radiation treatment--the results of Trans Tasman Radiation Oncology Group study TROG 99.02. Radiother Oncol. 2011 Jan;98(1):93-8. PubMed PMID: 21109321. English.

3. Leon O, Guren MG, Radu C, Gunnlaugsson A, Johnsson A. Phase I study of cetuximab in combination with 5-fluorouracil, mitomycin C and radiotherapy in patients with locally advanced anal cancer. Eur J Cancer. 2015 Dec;51(18):2740-6. PubMed PMID: 26597443. English.

4. El-Hadaad HA, Wahba HA, Roshdy S. Concomitant chemoradiotherapy with Cisplatin plus 5-Fluorouracil for anal squamous cell carcinoma. J Gastrointest Cancer. 2015 Jun;46(2):156-60. PubMed PMID: 25810165. English.

5. Matzinger O, Roelofsen F, Mineur L, Koswig S, Van Der Steen-Banasik EM, Van Houtte P, et al. Mitomycin C with continuous fluorouracil or with cisplatin in combination with radiotherapy for locally advanced anal cancer (European Organisation for Research and Treatment of Cancer phase II study 22011-40014). European journal of cancer (Oxford, England : 1990). 2009 2009;45:2782-91.

6. Koerber SA, Slynko A, Haefner MF, Krug D, Schoneweg C, Kessel K, et al. Efficacy and toxicity of chemoradiation in patients with anal cancer--a retrospective analysis. Radiation oncology (London, England). 2014 2014;9:113.

7. James RD, Glynne-Jones R, Meadows HM, Cunningham D, Myint AS, Saunders MP, et al. Mitomycin or cisplatin chemoradiation with or without maintenance chemotherapy for treatment of squamous-cell carcinoma of the anus (ACT II): a randomised, phase 3, open-label, 2 x 2 factorial trial. Lancet Oncol. 2013 May;14(6):516-24. PubMed PMID: 23578724. English.

8. World Health Organisation W. WHO Handbook for Reporting Results of Cancer Treatment. Geneva: World Health Organisation, 1979. Report No.

9. Peiffert D, Tournier-Rangeard L, Gérard J-P, Lemanski C, François E, Giovannini M, et al. Induction chemotherapy and dose intensification of the radiation boost in locally advanced anal canal carcinoma: final analysis of the randomized UNICANCER ACCORD 03 trial. Journal of Clinical Oncology. 2012 2012;30:1941–8.

10. Crehange G, Bosset M, Lorchel F, Dumas JL, Buffet-Miny J, Puyraveau M, et al. Combining cisplatin and mitomycin with radiotherapy in anal carcinoma. Diseases of the colon and rectum. 2007 2007;50:43-9.

11. Meulendijks D, Dewit L, Tomasoa NB, van Tinteren H, Beijnen JH, Schellens JH, et al. Chemoradiotherapy with capecitabine for locally advanced anal carcinoma: an alternative treatment option. British Journal of Cancer. 2014 Oct 28;111(9):1726-33. PubMed PMID: 25167226. Pubmed Central PMCID: PMC4453727. English.

12. Bartelink H RFEFRPBJFGDGPDvGMPM. Concomitant radiotherapy and chemotherapy is superior to radiotherapy alone in the treatment of locally advanced anal cancer: results of a phase III randomized trial of the European Organization for Research and Treatment of Cancer Radiotherapy and Gastrointestinal Cooperative Groups. Journal of clinical oncology. 1997;15(5):2040.

13. Glynne-Jones R, Meadows H, Wan S, Gollins S, Leslie M, Levine E, et al. EXTRA--a multicenter phase II study of chemoradiation using a 5 day per week oral regimen of capecitabine and intravenous mitomycin C in anal cancer. Int J Radiat Oncol Biol Phys. 2008 2008;72:119-26.

14. Kouloulias V, Plataniotis G, Kouvaris J, Dardoufas C, Gennatas C, Uzunoglu N, et al. Chemoradiotherapy combined with intracavitary hyperthermia for anal cancer: feasibility and long-term results from a phase II randomized trial. American journal of clinical oncology. 2005 2005;28:91-9.

15. Edelman S, Johnstone PA. Combined modality therapy for HIV-infected patients with squamous cell carcinoma of the anus: outcomes and toxicities. Int J Radiat Oncol Biol Phys. 2006 Sep 1;66(1):206-11. PubMed PMID: 16904522. English.

16. Ferrigno R, Nakamura RA, Dos Santos Novaes PE, Pellizzon AC, Maia MA, Fogarolli RC, et al. Radiochemotherapy in the conservative treatment of anal canal carcinoma: retrospective analysis of results and radiation dose effectiveness. Int J Radiat Oncol Biol Phys. 2005 Mar 15;61(4):1136-42. PubMed PMID: 15752894. English.

17. Ceresoli GL, Ferreri AJ, Cordio S, Villa E. Role of dose intensity in conservative treatment of anal canal carcinoma. Report of 35 cases. Oncology. 1998 1998;55:525-32.

18. Grabenbauer GG, Matzel KE, Schneider IH, Meyer M, Wittekind C, Matsche B, et al. Sphincter preservation with chemoradiation in anal canal carcinoma: abdominoperineal resection in selected cases?. Diseases of the colon and rectum. 1998 1998;41:441-50.

19. Flam M, John M, Pajak TF, Petrelli N, Myerson R, Doggett S, et al. Role of mitomycin in combination with fluorouracil and radiotherapy, and of salvage chemoradiation in the definitive nonsurgical treatment of epidermoid carcinoma of the anal canal: results of a phase III randomized intergroup study. Journal of Clinical Oncology. 1996 1996;14:2527–39.

20. Salama JK, Mell LK, Schomas DA, Miller RC, Devisetty K, Jani AB, et al. Concurrent chemotherapy and intensity-modulated radiation therapy for anal canal cancer patients: a multicenter experience. Journal of clinical oncology : official journal of the American Society of Clinical Oncology. 2007 2007;25:4581-6.

21. Lee WS, Chun HK, Lee WY, Yun SH, Yun H, Cho YB, et al. Anal canal carcinoma: experience from a single Korean institution. Yonsei Med J. 2007 Oct 31;48(5):827-32. PubMed PMID: 17963341. Pubmed Central PMCID: PMC2628150. English.

22. Martenson JA, Lipsitz SR, Wagner HJ, Kaplan EH, Otteman LA, Schuchter LM, et al. Initial results of a phase II trial of high dose radiation therapy, 5-fluorouracil, and cisplatin for patients with anal cancer (E4292): an Eastern Cooperative Oncology Group study. Int J Radiat Oncol Biol Phys. 1996 1996;35:745-9.

23. Wagner JP, Mahe MA, Romestaing P, Rocher FP, Berger C, Trillet- Lenoir V, et al. Radiation therapy in the conservative treatment of carcinoma of the anal canal. International Journal of Radiation Oncology Biology Physics. 1994;29(1):17-23. PubMed PMID: 1994146153. English.

24. Sandhu AP, Symonds RP, Robertson AG, Reed NS, McNee SG, Paul J. Interstitial iridium-192 implantation combined with external radiotherapy in anal cancer: ten years experience. Int J Radiat Oncol Biol Phys. 1998 1998;40:575-81.

25. Hwang JM, Rao AR, Cosmatos HA, Wang R, Kaptein JS, Kagan RA, et al. Treatment of T3 and T4 anal carcinoma with combined chemoradiation and interstitial 192Ir implantation: a 10-year experience. Brachytherapy. 2004 2004;3:95-100.
